# Integration of Cross Species RNA-seq Meta-Analysis and Machine-Learning Models Identifies the Most Important Salt Stress–Responsive Pathways in Microalga *Dunaliella*

**DOI:** 10.3389/fgene.2019.00752

**Published:** 2019-08-29

**Authors:** Bahman Panahi, Mohammad Frahadian, Jacob T. Dums, Mohammad Amin Hejazi

**Affiliations:** ^1^Department of Genomics, Branch for Northwest & West region, Agricultural Biotechnology Research Institute of Iran (ABRII), Agricultural Research, Education and Extension Organization (AREEO), Tabriz, Iran; ^2^Department of Animal Science, Faculty of Agriculture, University of Tabriz, Tabriz, Iran; ^3^Department of Plant and Soil Sciences, University of Delaware, Newark, DE, USA; ^4^Department of Food Biotechnology, Branch for Northwest & West region, Agricultural Biotechnology Research Institute of Iran (ABRII), Agricultural Research, Education and Extension Organization (AREEO), Tabriz, Iran

**Keywords:** Dunaliella, RNA-seq meta-analysis, machine learning, network, retrograde signaling, ROS, tetrapyrrole

## Abstract

Photosynthetic microalgae are potentially yielding sources of different high-value secondary metabolites. Salinity is a complex stress that influences various metabolite-related pathways in microalgae. To obtain a clear view of the underlying metabolic pathways and resolve contradictory information concerning the transcriptional regulation of *Dunaliella* species in salt stress conditions, RNA-seq meta-analysis along with systems levels analysis was conducted. A p-value combination technique with Fisher method was used for cross species meta-analysis on the transcriptomes of two *Dunaliella salina* and *Dunaliella* *tertiolecta* species. The potential functional impacts of core meta-genes were surveyed based on gene ontology and network analysis. In the current study, the integration of supervised machine-learning algorithms with RNA-seq meta-analysis was performed. The analysis shows that the lipid and nitrogen metabolism, structural proteins of photosynthesis apparatus, chaperone-mediated autophagy, and ROS-related genes are the keys and core elements of the *Dunaliella* salt stress response system. Cross-talk between Ca^2+^ signal transduction, lipid accumulation, and ROS signaling network in salt stress conditions are also proposed. Our novel approach opens new avenues for better understanding of microalgae stress response mechanisms and for selection of candidate gene targets for metabolite production in microalgae.

## Introduction

Microalgae are photosynthetic organisms that are considered potential sources of different secondary metabolites such as β-carotene and lipid (Alcantara et al., 2013; Klein et al., 2013). Microalgae produce these metabolites by harvesting sunlight and subsequently fixing CO_2_ using this energy. It has been proposed that efficiency of CO_2_ fixation and consequently the production rate of lipids and secondary metabolites are affected by different stresses such as salt, light, temperature, pH, and nutrient starvation (Takagi, 2006; Devi and Venkata, 2012). These are common stresses found in industrial production of microalgae and are usually considered to hamper production. In general, stress decreases the microalgae growth rate and biomass production, although it is well known that several stresses can be used to increase lipid and/starch accumulation; however, the increased accumulation per cells does not often make up for the lost cellular growth. Although attempts have been made to manipulate the stress response; however, progress has been limited due to the lack of understanding of the basic metabolism of algae and how the different stresses impact metabolic pathways (Shin et al., 2015).

It has been reported that salt stress induce the glycerol metabolism enzymes such as glycerol-3-phosphate phosphatase (GPP), glycerol 2-dehydrogenase (NADP^+^) (DHAR), and dihydroxyacetone kinase (DHAK) activity in *Dunaliella salina* (Breuer et al., 2013). Similar results have been obtained on enzymatic activities of fructose-bisphosphate aldolase (FBPA) involved in starch metabolisms (Klok et al., 2013). It has been noted that the enzymatic activities of ribulose-5-phosphate kinase (RuPK), ribulose-bisphosphate carboxylase (RuBisCO), phosphoglycerate kinase (PGK), and glyceraldehyde-3-phosphae dehydrogenase (GAPDH) involved in photosynthetic carbon fixation increase in stress condition (Ben‐Amotz, 1975; Beardall et al., 1976; Johnson et al., 1976;Wegmann, 1979).

Moreover, transcriptional regulation of metabolic enzymes is closely associated with the growth rate and physiological conditions (Brauer et al., 2008). So, stress-responsive transcripts can be populating with the slow growth and metabolite production.

It has been proposed that the transcription of enzymes involved in glycerol metabolisms and its potential carbon sources increases under salinity stress condition. Moreover, correlated transcriptional regulation of enzymes involved in glycerol metabolisms with the flow of pathways has been proposed (Fang et al., 2017). Transcriptomic study of *Klebsormidium crenulatum* has showed increase of sucrose synthase, sucrose phosphate synthase, and several enzymes involved in the biosynthesis of the raffinose family of oligosaccharides after desiccation stress (Holzinger et al., 2014).

However, literatures have showed contradictory findings about transcriptional regulation (Alkayal et al., 2010; Cui et al., 2010; Kim et al., 2010). These incongruences are mostly related to differences in severity, time range of treatments, and sample size (Farhadian et al., 2018b).

Due to the extensive application of RNA-seq technology for global expression analysis, the amount of deposited transcriptome data in stress condition is exponentially increasing. With the considerable increasing of deposited transcriptome data for the various physiological conditions, generalization of the major transcriptome regulatory mechanism is essential to provide meaningful and precise biological conclusions.

It has been proposed that combining the results of independent studies with meta-analysis can bypass the challenges associated with individual transcriptome studies (Sharifi et al., 2018). In the previous meta-analysis studies, differentially expressed genes (DEGs) involved in multiple stresses were identified (Ashrafi-Dehkordi et al., 2018). Kong et al. (2019) investigated a common transcriptional response to salt stress in different rice genotypes at the seedling stage. Wang et al. (2018a), Wang et al. (2018b) also identified the salt stress responding genes using transcriptome analysis in green algae *Chlamydomonas reinhardtii* and *Dunaliella salina,* respectively.

In the current study, for the first time, we integrated RNA-seq meta-analysis and supervised machine-learning models to detect and prioritize the salt stress responding genes and pathways which held common between two *Dunaliella tertiolecta* and *D. salina* species. Machine learning is the term of computer science in which computational statistics and information theory employ to construct algorithms that can learn from data (Wang et al., 2018a). The learning process refers to knowledge discovery that translate the features in the existing data sets into pattern (Yu et al., 2018). Machine learning has attracted wide attention for its various applications in modern biology such as cancer study (Akay, 2009), robust phenotyping (Platt, 1999), and transcriptome data analysis (Ebrahimi et al., 2014). Guo et al. (2016) applied the MinReg algorithm to infer the global gene regulatory networks in *Fusarium graminearum* on transcriptome datasets. Moreover, machine learning–based differential network analysis has been applied to predict stress-responsive genes (Wang et al., 2018a). Moreover, feasibility of supervised machine-learning models on bio-signature identification has been confirmed by Farhadian et al. (2018a) and Sharifi et al. (2018). We used various feature selection algorithms for modeling and ranking of common stress responding genes and proposed some important salt stress–responsive genes and pathways in two species of *Dunaliella* microalga.

## Methods and Materials

### Data Set Collection

RNA-seq raw reads were retrieved from the European Nucleotide Archive database. One *D. salina* and two *D. tertiolecta* datasets were selected for meta-analysis. The first dataset from *D. tertiolecta* (PRJNA385719) contains six biological samples which were grown in 0.08 M NaCl–treated ATCC media, harvested during stationary phase, and sequenced using Illumina MiSeq platform. The second dataset from *D. tertiolecta* (PRJNA51835) had five biological samples that were grown in 0.5 M NaCl were sequenced using Illumina GAIIx platform. The third dataset (PRJNA295823) contains reads from 18 salt–treated samples of *D. salina*. In this dataset, cells were grown in 0.5 M and harvested during stationary phase of growth for sequencing with Illumina HiSeq 2000 platform. In this work, samples that were treated with high salinity were included in our analysis.

### RNA-seq and Differential Gene Expression Analysis

FastQC v0.11.5 (http://www.bioinformatics.babraham.ac.uk/projects/fastqc/) was used to assess quality of datasets, and reads were trimmed using Trimmomatic v0.32 (Bolger et al., 2014). The filtered reads were *de novo* assembled using Trinity v2.4.0 (Haas, 2013). The Trinity was run-in strand-specific mode (using the “—SS_lib_type RF” and “—SS_lib_type FR” options for *D. tertiolecta and D. salina* detests, respectively). Filtered reads from each biological sample were aligned to the *de novo* assembled transcripts using Kallisto (v0.44.0) with default parameters. Reads abundant per each transcript were normalized using fragment per kilo bases per million (FPKM), and the deferentially expressed genes (assembled transcripts) between treated and untreated samples were captured using Fisher model in edgeR package (Robinson et al., 2010). Significant differential expression was defined as a fold change ≥ |2| and a false discovery rate (FDR) corrected p-value ≤ 0.05 (Benjamin and Hochberg, 1995).

### Orthology Definition and Meta-Analysis

Protein orthology was determined using Blastx (cutoff value of 6) against *C. reinhardtii*, *Volvox carteri*, and *D. salina* (https://phytozome.jgi.doe.gov/). The best hits were extracted with an in-house python scripts (Supplementary script S1). A meta-analysis was carried out on the integrated dataset to find the DEGs between two species. First, to reduce number of statistical tests and control of false positives, 10% of genes that have low expression levels and variance were excluded. A comparison between two classes for each species designed and moderated t-statistic with 1,000 random permutations carried out to define the genes with significant expression. The adjusted p-value (FDR <0.05) (Benjamini and Hochberg, 1995) were considered significant. P-value of DEGs in the each of the datasets was merged. To combine p-values of DEGs between two conditions, Fisher method was used. The log ratio of means (ROM) was applied to measure the gene expression values by following formula:

$$y_{gn}=ln\left[\frac{{\bar{r}}_{gr}}{{\bar{r}}_{gs}}\right]$$

where $y_{gn},{\bar{r}}_{gr},{\bar{r}}_{gs}$ represent ROM, mean expression level for each gene in dataset, respectively. The preprocessing and analysis were performed with the metaRNASeq package (Rau et al., 2014) of R software. A Venn diagram was generated using the ggplot2 package in R (Wickham, 2016).

### Gene Ontology Enrichment and Functional Analysis

GO enrichment analysis of biological process (BP), molecular function (MF), and cellular component (CC) categories with p-value < 0.05 cutoff was performed using the Algal Functional Annotation tools (Lopez et al., 2011). Pathway enrichment of DEGs and meta-analysis results were visualized in MapMan software (Thimm, 2004).

### Protein–Protein Network Analysis

Protein function information is critical for the elucidation of dynamics in complex processes (Panahi et al., 2014b; Panahi et al., 2015). This study used STRING database version 11.0 (https://string-db.org/) to predict protein–protein interactions networks from the DEGs. The k-means clustering algorithm was used for the functional module identification. Finally, identified modules were enriched using the KEGG database version 88.2.

### Supervised Machine-Learning Models

Data cleaning on the merged dataset was conducted with useless and correlated attribute-removing approaches. The useless and correlated attributes (genes) were defined for genes with expression variation lower than 0.1 and correlation higher than 95%, respectively. Cleaned data subsequently were normalized, and the results from different weighting algorithms were presented as values between 0 and 1. Different attribute weighting algorithms including the information gain, information gain ratio, chi-squared, deviation, rule, SVM, Gini index, uncertainty, and relief were used as supervised machine-learning models to repeat ably investigation of the discrimination genes between the control and stress conditions in the *Dunaliella* spp. Two approaches were used to survey the species dependency or independency of identified meta-genes. For the first approach, models were run for each separate species while the stress treatment status was defined as a label variable. Discriminating genes that were shared by both species were defined as species-independent salt stress–responsive genes. In the second approach, the expression value (count data) and type of species (*D. salina* and *D. tertiolecta*) were set as features for attribute weighting while stress treatment status was defined as a label variable. The importance value of each feature calculates as (1-*p*) where *p* was the *p*-value of the feature selection test between the candidate predictor and the stress condition.

## Results

### *De Novo* Assembly

Strand-specific RNA sequencing data from each condition were pooled together for *de novo* assembly and subsequent gene expression analysis. In PRJNA385719 data set, 17,312 transcripts were matched to proteins based on our criteria. Moreover, transcript length ranged from 110 bps to 15,458 bps. Detailed assembly information of three data sets was provided in **Table 1**.

**Table 1:** Read and assembly statistics of datasets.

	Total raw reads	Total processed reads	Number of coting’s	GC (%)	Size range (pb)
PRJNA385719	40,868,954	3,452,785	17,312	50	110-15,458
PRJNA51835	41,635,032	4,645,287	17,856	49	115-14,751
PRJNA295823	38,475,935	3,124,575	16,957	50	114-14,971

### Metabolic Overview of Differentially Expressed Genes

**Figure 1:**
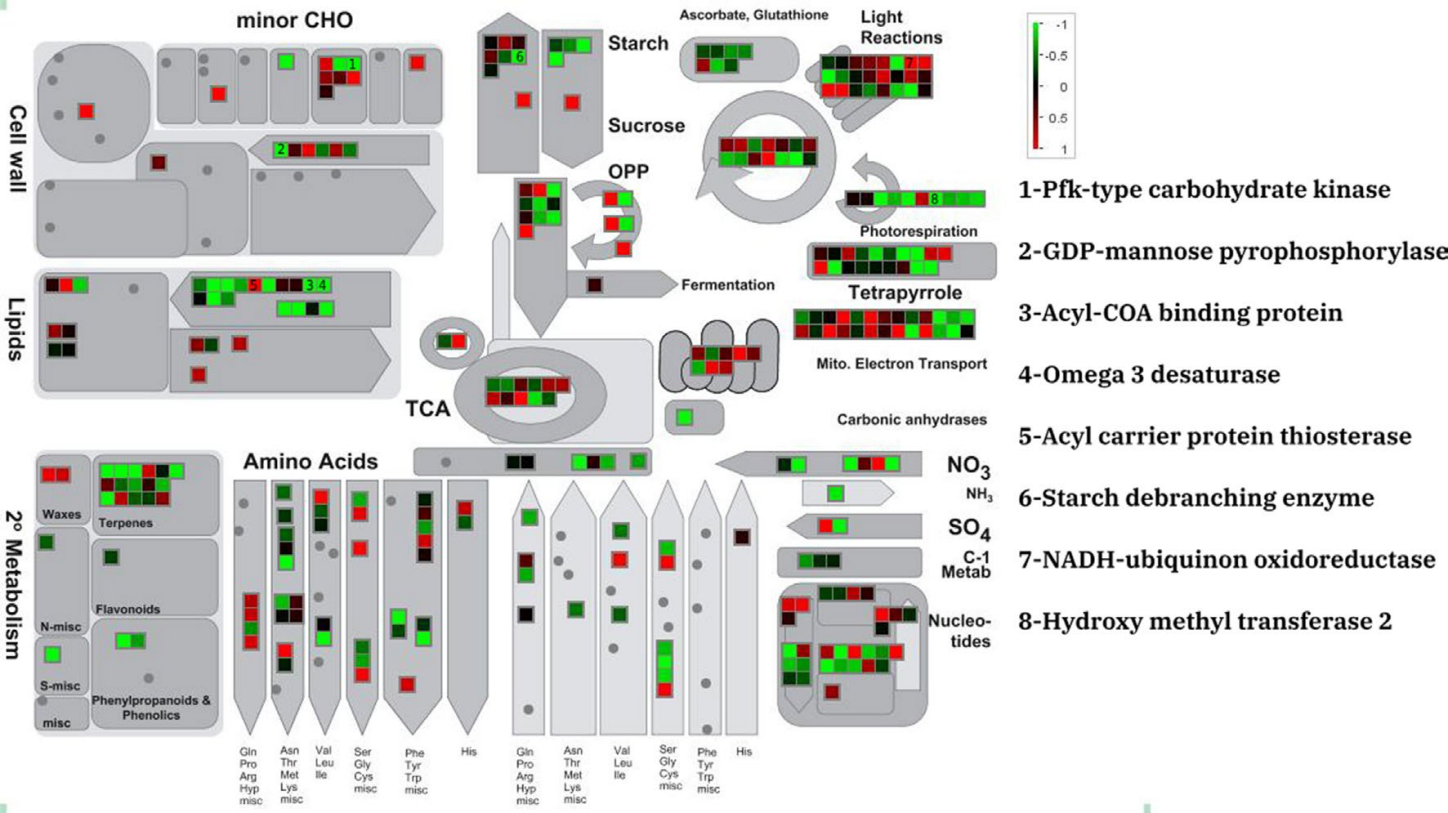
Metabolic overview of differentially expressed genes of *D. tertiolecta* (PRJNA51835) in responses to salt stress.

The MapMan annotation tool was used to display potential metabolic impacts from DEGs the three different data sets (**Figure 1** and **Tables S1** **–** **S3**). DEGs were annotated as minor carbohydrate, light reactions, sucrose and starch, lipid, amino acid, and TCA metabolism. The three experiments showed similar expression patterns for the metabolic genes although the amount of expression was different. For example, a putative PfkB-type carbohydrate kinase which participate in minor carbohydrate metabolism showed severe (fold change > 3), moderate (2 < fold change < 3), and lower (2 > fold change) down-regulation in PRJNA385719, PRJNA51835, and PRJNA295823, respectively. Of all the lipid metabolism genes, an acyl carrier protein thioesterase was dramatically up-regulated in all experiments. This is contrast to majority of lipid metabolism genes that were moderately down-regulated in the salt stress condition. Species-specific patterns were observed for the light reaction genes. In *D. tertiolecta* datasets, the moderate up- and down-regulated genes were uniformly observed whereas in *D. salina* dataset; most of light reactions underlying genes were moderately down-regulated in salt stress condition.

### RNA-seq Meta-Analysis

**Figure 2:**
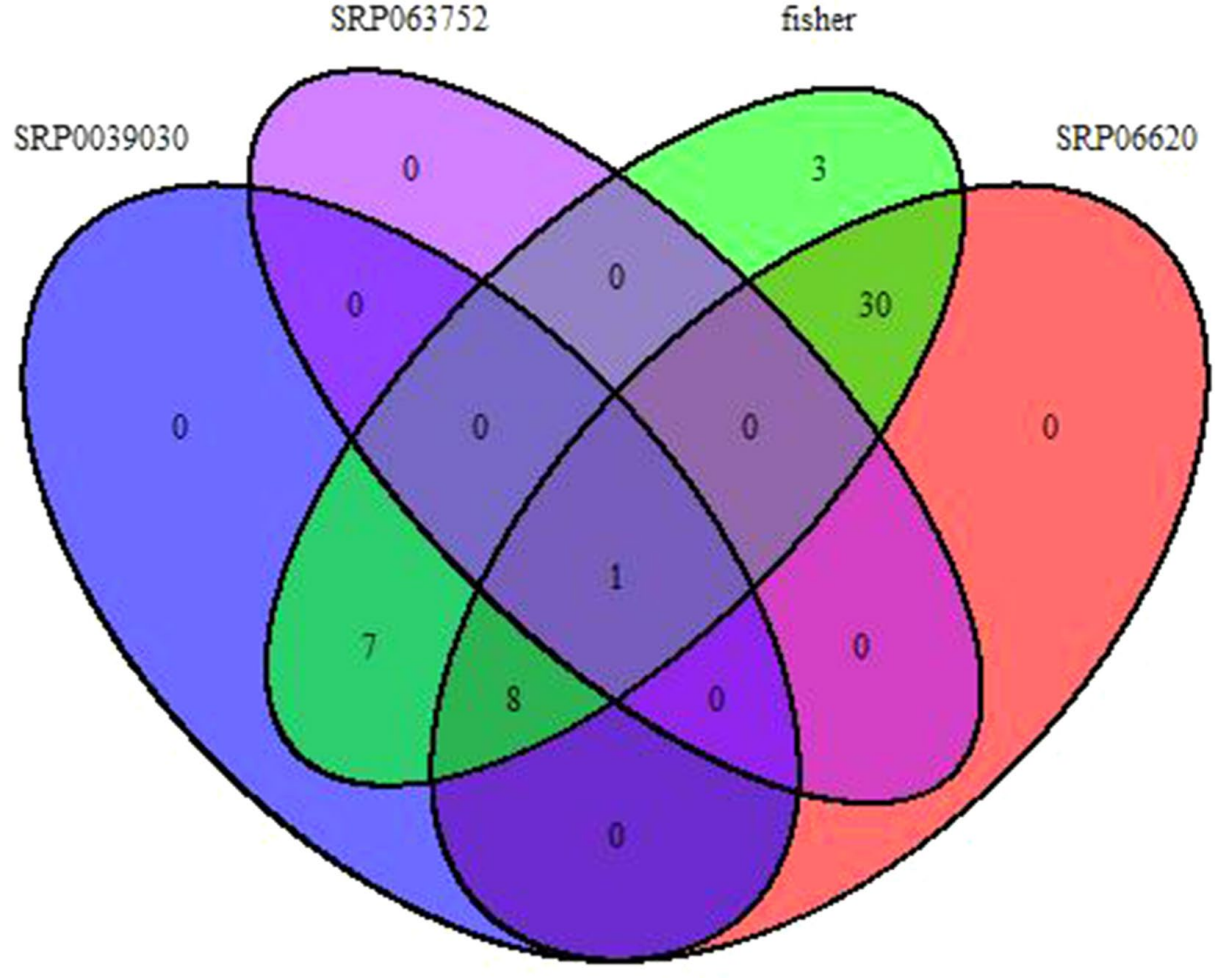
Venn diagram of identified meta-genes in three data sets based on Fisher method.

**Figure 3:**
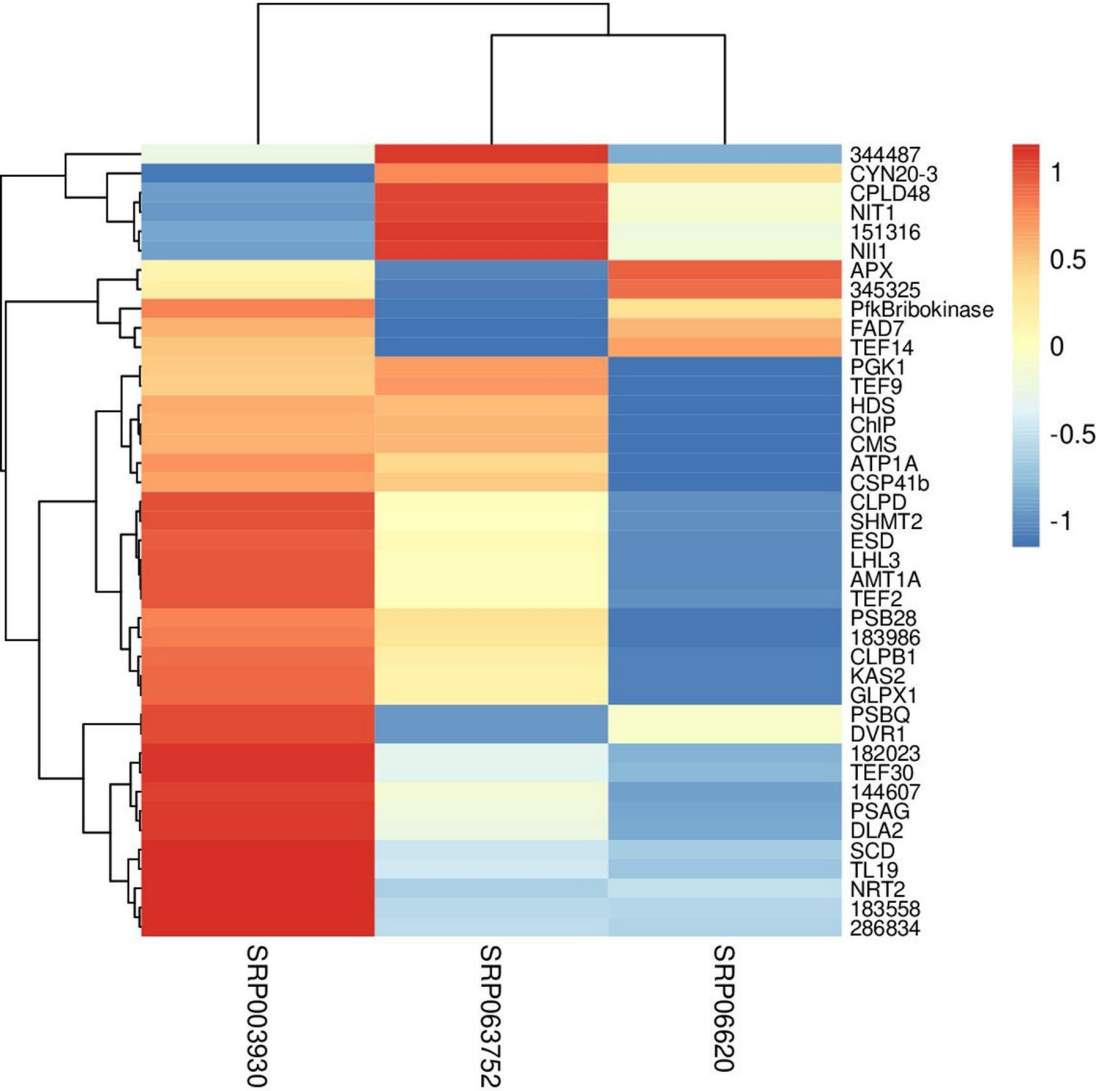
Clustering of metagenes based on expression patterns in three data sets. The fold changes were used as the expression value in constructing heatmap.

Fisher method defined 49 differentially expressed transcripts representatives of 41 meta-genes (**Figure 2**). Details of identified meta genes and annotations were presented in **Table 2**. Of the 41 meta-genes, AMT1A, CLPD, and CLPB1, which encode ammonium transporter, chloroplast ClpD chaperone, and cytosolic ClpB chaperone, respectively, were up-regulated in salt stress conditions (**Figure 3**).

**Table 2:** Detailed information of identified meta genes and corresponding annotations.

Protein ID (JGI v4.0 ID)	Annotated name	DefLine
55268	HDS	1-hydroxy-2-methyl-2-(E)-butenyl 4-diphosphate synthase, chloroplast precursor
56237	FAD7	Chloroplast glycerolipid omega-3-fatty acid desaturase
76602	ATP1A	Mitochondrial F1F0 ATP synthase, alpha subunit
111372	PfkB	PfkB-type carbohydrate kinase
132210	PGK1	Phosphoglycerate kinase
135322	CSP41b	Chloroplast stem-loop-binding protein
136810	ChlP	Geranylgeranyl reductase
139619	KAS2	3-ketoacyl-ACP-synthase
150826	TEF9	Predicted protein
152648	CPLD48	Predicted protein
153656	PSBQ	Oxygen evolving enhancer protein 3
158745	AMT1A	Ammonium transporter
159574	GLPX1	Fructose 1,6-bisphosphatase
165416	PSAG	Photosystem I reaction center subunit V
175746	ESD	Esterase D
182361	TEF14	Thylakoid lumenal protein
182896	PSB28	Photosystem II subunit 28
184661	NIT1	Nitrate reductase
185309	LHL3	Low molecular mass early light-induced protein
185571	CYN20-3	Peptidyl-prolyl cis-trans isomerase, cyclophilin-type
192085	NII1	Nitrite reductase
194676	TEF2	Rhodanese-like Ca-sensing receptor
195417	CLPD	ClpD chaperone, Hsp100 family
195423	CLPB1	ClpB chaperone, Hsp100 family
195952	DVR1	3,8-divinyl protochlorophyllide a 8-vinyl reductase
196354	SHMT2	Serine hydroxymethyltransferase 2
196500	DLA2	Dihydrolipoamide acetyltransferase
196604	CMS	4-diphosphocytidyl-2C-methyl-D-erythritol synthase, chloroplast precursor
205649	TL19	Thylakoid lumen protein
205993	TEF30	Predicted protein
206548	APX	L-ascorbate peroxidase
345325	NAR3	Nitrate/nitrite transporter
117883	SCD	Stearoyl-CoA desaturase ∆9
144607		
151316	WD40	WD40 repeat-like superfamily protein
182023	hypothetical protein	
183558	DUF	Containing domain of unknown function (DUF4399)
183986	LHCA2	Chlorophyll A-B binding protein
192088	NAR4	High affinity nitrate transporter (system II)
286834	F4J9G2	Rhodanese/Cell cycle control phosphatase superfamily protein
344487	hypothetical protein	

### Functional Impacts of Meta-Genes Based on Gene Ontology and Network Analysis

Functional gene ontology analysis of identified meta-genes was conducted in three categories including biological process (BP), MF, and CCs (**Table 3**). In the biological process, fatty acid and carboxylic acid biosynthetic processes were enriched (**Table 3**). Regarding the MF categories, oxidoreductase activity was most prevalent, even though different functions such as CoA desaturase, fatty acid synthase, omega-3 fatty acid desaturase, stearoyl-CoA 9-desaturase, nitrate reductase (NADH), ferredoxin-nitrite reductase, and geranylgeranyl reductase activities were also enriched (**Table 3**).

**Table 3:** Gene ontology enrichments of meta genes in three categories including BP (biological process), MF (molecular functions) and CC (cellular components), number of hits, and corresponding FDR value.

GO names	GO ID	GO category	Hits	FDR
Fatty acid biosynthetic process	GO:0006633	BP	3	0.000175
Lipid biosynthetic process	GO:0008610	BP	3	0.001142
Photosynthesis	GO:0015979	BP	2	0.001301
Nitrate assimilation	GO:0042128	BP	1	0.002636
Carboxylic acid biosynthetic process	GO:0046394	BP	3	0.004328
D-ribose metabolic process	GO:0006014	BP	1	0.00789
Small molecule biosynthetic process	GO:0044283	BP	4	0.009843
Monosaccharide metabolic process	GO:0005996	BP	2	0.010695
Pentose metabolic process	GO:0019321	BP	1	0.015722
Chlorophyll biosynthetic process	GO:0015995	BP	1	0.01832
Alcohol metabolic process	GO:0006067	BP	2	0.020593
Cellular carbohydrate metabolic process	GO:0044262	BP	2	0.02434
Oxygen and reactive oxygen species metabolic process	GO:0072593	BP	1	0.026077
Superoxide metabolic process	GO:0006801	BP	1	0.026077
Oxoacid metabolic process	GO:0043436	BP	3	0.035025
Cellular ketone metabolic process	GO:0042180	BP	3	0.035662
Ferredoxin-nitrite reductase activity	GO:0048307	MF	1	0.002307
Geranylgeranyl reductase activity	GO:0045550	MF	1	0.002307
3,4-dihydrocoumarin hydrolase activity	GO:0018733	MF	1	0.004609
1-oxa-2-oxocycloheptane lactonase activity	GO:0018731	MF	1	0.004609
Butyrolactone hydrolase activity	GO:0018734	MF	1	0.004609
Nitrate reductase (NADH) activity	GO:0009703	MF	1	0.004609
Phosphoglycerate kinase activity	GO:0004618	MF	1	0.004609
Stearoyl-CoA 9-desaturase activity	GO:0004768	MF	1	0.004609
Sulfolactone hydrolase activity	GO:0018732	MF	1	0.004609
Ribokinase activity	GO:0004747	MF	1	0.006906
3-oxoacyl-[acyl-carrier-protein] synthase activity	GO:0004315	MF	1	0.009198
Oxidoreductase activity	GO:0016491	MF	5	0.009936
CoA desaturase activity	GO:0016215	MF	1	0.016044
Omega-3 fatty acid desaturase activity	GO:0042389	MF	1	0.016044
Phosphotransferase activity, carboxyl group as acceptor	GO:0016774	MF	1	0.022847
Fatty acid synthase activity	GO:0004312	MF	1	0.031849
Dioxygenase activity	GO:0051213	MF	1	0.034088
Phospholipase activity	GO:0004620	MF	1	0.034088
Inorganic anion transmembrane transporter activity	GO:0015103	MF	1	0.042994
Oxygen evolving complex	GO:0009654	CC	1	0.012942
Extrinsic to membrane	GO:0019898	CC	1	0.015086
Membrane part	GO:0044425	CC	3	0.021468
Endoplasmic reticulum	GO:0005783	CC	1	0.021495
Thylakoid part	GO:0044436	CC	1	0.036321

**Figure 4:**
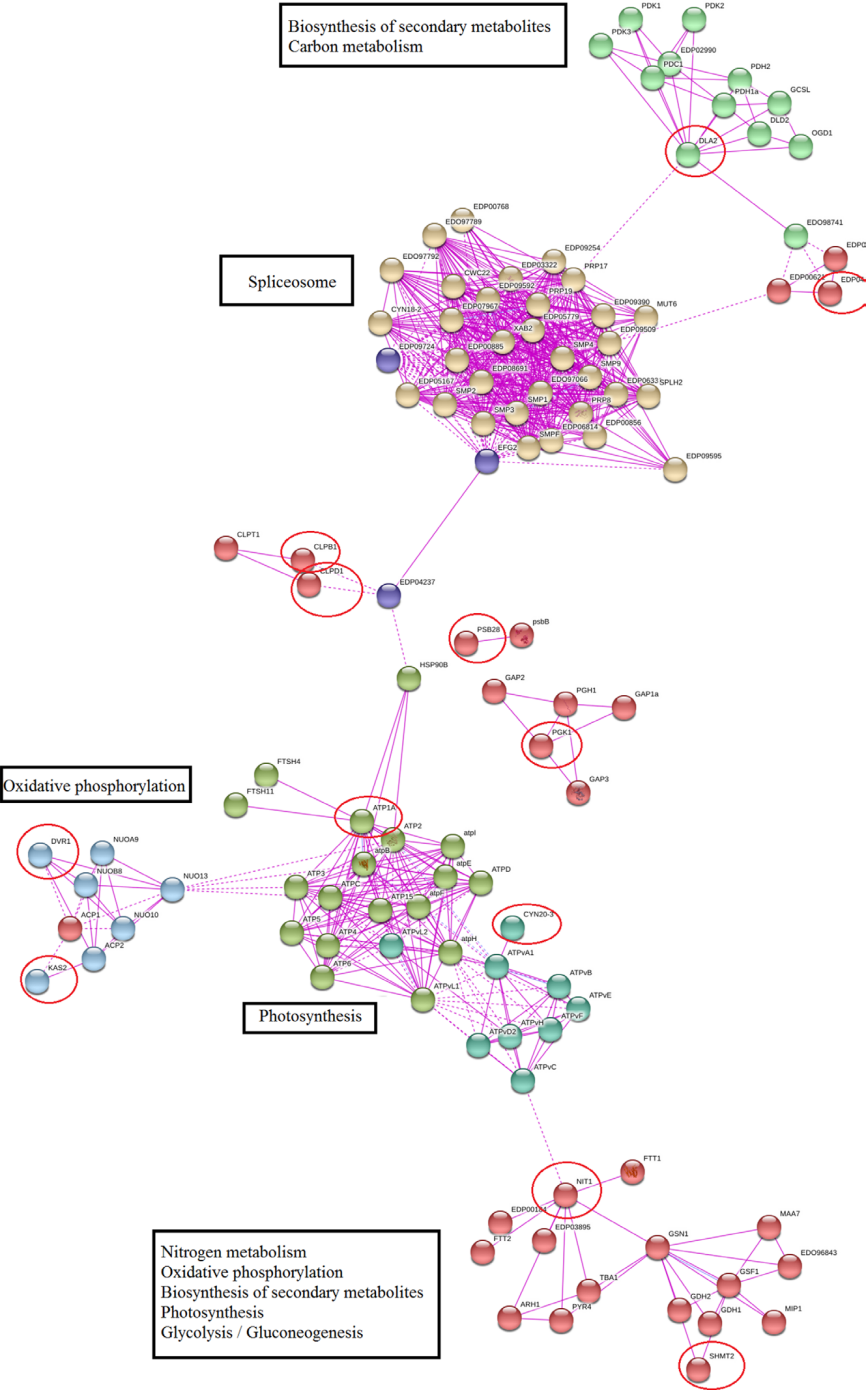
Protein–protein interaction network of meta-genes. The unconnected meta-genes were removed from constructed network. Meta-genes were signed by red circles.

Protein–protein network of meta-genes based on co-expression and experimentally verified knowledge showed that 60% of identified meta-genes had a significant interaction with important functional modules, and remaining meta-genes had no other connections in the network (these nodes were removed from constructed network). Nitrogen metabolism, photosynthesis, oxidative phosphorylation, and splicing were the most important modules in the constructed network (**Figure 4**). We used a network modules analysis to investigate the core molecular networks that may be participating in biosynthesis of secondary metabolisms. Closer inspection of constructed networks revealed some important finding in *Dunaliella* responses to salt stress including 1) SHMT2 as important coordinator between nitrogen and carbon metabolism, photosynthesis, and secondary metabolite biosynthesis; 2) crosstalk between identified functional modules and splicing as a transcriptome plasticity mechanism; 3) anterograde-/retrograde-signaling networks importance in *Dunaliella* responses to salt stress condition; and 4) crosstalk between tetrapyrrole and secondary metabolite biosynthesis.

### Data Mining

Two hundred ninety-six attributes were selected from 2,900 common genes of merged file after data cleaning steps. The attributes with weight values higher than 0.5 were selected (**Table S4**). Results of species-specific analysis were also presented in **Tables S5** and **S6**. Of the 41 meta-genes, 16 genes were selected by more than three weighting algorithms (**Table 4**). The verified meta-genes were related to photosynthesis (PSBQ, LHL3), lipid metabolism (ESD, KAS2), nitrogen metabolism (NIT1), ROS detoxification (APX, SHMT2), and retrograde-signaling network (DVR1, LHL3). Thereafter, the verified genes and pathways were defined as core and key salt stress–responsive genes and pathways in *Dunaliella*.

**Table 4:** Machine learning models based on attribute weighting algorithms demonstrated the most important salt stress responsive genes (species independent).

Attribute	The number of weighting models
PSBQ	5
SCD	5
GLPX1	4
PSAG	4
ESD	4
LHL3	4
APX	4
PfkB	3
CSP41b	3
ChlP	3
KAS2	3
NIT1	3
DVR1	3
SHMT2	3

## Discussion

Recently, high-throughput transciptomics data has helped increase the elucidation of the complexity of gene regulation in various abiotic stress conditions (Panahi et al., 2014b; Panahi et al., 2015). However, the complex interaction between genes and environment is not yet well understood. It has been proposed that integrative analysis of global gene expression data is effective approach for identification of key regulatory networks (Panahi et al., 2013; Shahriari Ahmadi et al., 2013; Farhadian et al., 2018a; Panahi et al., 2019). To our knowledge, this is the first study where multiple transcriptomic datasets under salt stress condition were used to probe the genetic response of the *Dunaliella* spp. In the current study, integration of supervised machine-learning algorithms with RNA-seq meta-analysis was proposed that lipid and nitrogen metabolism, structural proteins of photosynthesis apparatus, signaling, and ROS-related genes are the key and core elements of the *Dunaliella* salt stress response system.

### Photosynthesis Machinery Structural Proteins as Important Salt Stress–Responsive Genes

Photosynthesis–related structural and functional proteins such as chloroplast stem-loop–binding protein (CSP41b), oxygen-evolving enhancer protein (PSBQ), photosystem II reaction center protein (PSB28), photosystem I reaction center subunit V (PSAG), thylakoid luminal protein (TEF14), and photosystem I chlorophyll a–/b–binding protein 2 (LHCA2) were all defined as meta-genes. These findings of the current study are consistent with those of Ji et al. (2018) who found that photosystem II (PSII) is one of the most sensitive components of the electron transport chain under stress condition (Ji et al., 2018). So, the presence of several photosystem structural genes as meta-genes in salt stress is not unsurprising; more importantly, some of these genes (PSBQ and PSB28) were defined as key salt stress–responsive genes (**Table 2**). The PSBQ protein is an extrinsic subunit of the PSII and is necessary for the regulation of both activity and assembly of PSII (Thornton et al., 2004; Summerfield et al., 2005). The down-regulation of PSBQ during salt stress in the three datasets also agrees with a previous study done on other *Dunaliella* spp. (Suorsa et al., 2006). The importance of PSBQ transcriptional in response to salt stress in *Dunaliella* was also confirmed by five different machine-learning algorithms (**Table 4**). Another PSII-related gene, PSB28 was also an important meta-genes for *Dunaliella* spp. PSB28 is involved in the biogenesis of PSII inner antenna CP47 (PsbB) and is essential for the protection of the reaction-center against high-light stress (Weisz et al., 2017). Our data suggests that PSB28 may also play a role in the salt stress response. The down-regulations of PSBQ and PSB28 may be an important adaptation response for microalgae against salt stress. In addition to PSII, photosystem I (PSI) was also affected by salt stress. PSI is composed of chlorophyll-binding core complex and a chlorophyll a–/b–binding peripheral antenna called light harvesting complex (LHCs). The results of transcriptome meta-analysis along with machine-learning weighting confirmed the importance of PSAG and LHCA2 in adaptation responses to salt stress condition (**Table 2**). It has been proposed that salt stress weakens the connection between LHCs and PSI and consequently reduces the conversion of light energy to chemical energy (Gupta et al., 2017). Our hypothesis has been also confirmed by recent study (Wang et al., 2019). Wang et al. (2019) found that salt stress induce protein interactions between FTSY-RPL13a-RPL18-EIF3A and chlL-chlN-rbcL-psaB-psaA-LHCB4-ATPvL1-atpI-cox1. The downregulation of rbcL, HSP90A, and LHC in the PPI network was also consistent with previous findings (Wang et al., 2019). It has also been found that chlorophyll a–/b–binding proteins such as LHCA2 are affected by light, oxidative stress, and chlorophyll retrograde signaling (Gupta et al., 2017). Downregulation of LHC under the stress condition corroborates these earlier finding that downregulation of the LHC under stress conditions is an attempt to minimize energy utilization by lowering photosynthetic demands (Xu et al., 2012). It has been proposed that these down regulations are attempting to minimize energy utilization by lowering photosynthetic demands. Additionally, decreased levels of chlorophyll a–/b–binding proteins were correlated with accumulation of ROS (Xu et al., 2012). Our data also confirms the coordinate response of chlorophyll a–/b–binding proteins, signaling, and ROS detoxification system–related genes (**Tables 2** and **4** and **Figure 4**).

### Contribution of ROS Scavenging and Signaling Pathways in Adaptation Network

In the present study, several meta-genes (APX, CLPB1, CLPD, LHL3, SHMT2, DVR1, and WD40, which encode ascorbate peroxidase, chaperone protein ClpB1 chaperone protein ClpD, Lhc-like protein, serine hydroxyl methyl transferase, protein DVR-1, and WD40 repeat-containing protein, respectively) were found as the main backbone of ROS and signaling network. Although different scavenging enzymes were up-regulated in response to salt stress, APX was the only enzyme selected as meta-genes in *Dunaliella* (**Figure 1** and **Table 2**) and also verified by four machine learning–based weighting algorithms (**Table 4**). This may indicate that APX is more effective than other scavenging enzymes. Although there are no published reports comparing the efficiency of different algal scavenging enzymes in salt stress conditions, it has been reported that APX activity in halophyte plants is more important than other scavenging enzymes (Shalata et al., 2001; Sekmen et al., 2007; Panahi et al., 2014a). Due to the dual roles of ROS in toxicity and as signal molecules, *Dunaliella* species seems to have developed complex strategies to regulate and detoxify ROS in salt stress conditions. Meta-analysis and machine learning–based weighting algorithms analysis proposed that chaperone-mediated autophagy (CMA) is another important system for *Dunaliella* spp. to cope with salt stress conditions (Xiong et al., 2007).

CLPB1 and CLPD and DVR1 are other groups of important salt stress–responsive genes in *Dunaliella*. These chaperones are proposed to be involved in plastid protein quality control and degradation of oxidized proteins (Ramundo et al., 2014).

SHMT1 (serine hydroxyl methyl transferase 1), which regulates ROS generation by controlling photorespiratory pathways, was another important ROS signaling–related genes (Moreno et al., 2005). SHMT1 is known to influence resistance to different stress conditions and mutation of SHMT1 resulted in increased cell damage due to strong accumulation of H_2_O_2_ (Moreno et al., 2005). LHL3 (low molecular mass early light-induced protein) is proposed as an ROS protection system against oxidative damage and was identified as a meta-gene for *Dunaliella* spp. (Hutin et al., 2003). Additionally, the presence of spliceosome components and ROS signaling cascades in the meta-genes suggests cross-talk between these pathways (**Figure 4**), and this is reflected in a recent investigation showing that spliceosomal protein mutants are related with ROS accumulation (Gu et al., 2018).

### Cross-Talk Between ROS Signaling Pathways, Lipid Biosynthesis, and Calcium Signal Transduction

Multiple studies reported that stress-induced lipid accumulation always correlates with an increase in antioxidant defenses systems (Hu et al., 2008; Zhao et al., 2015). In addition to their function in carbon and energy storage, lipids may act as antioxidants or protective defense molecules as part of the stress response (Hu et al., 2008). Our data also suggests this, since lipid metabolism–related genes responded transcriptionally to salt stress treatments in both species of *Dunaliella* (**Figure 1**). Particularly, KAS2 (3-ketoacyl-ACP-synthase) and FAD7 (chloroplast glycerol lipid omega-3-fatty acid desaturase) are implicated in the salt-induced response of lipid metabolism plasticity (**Tables 2** and **4**).

TEF2 which encodes a rhodanese-like Ca-sensing receptor was determined as another important gene in *Dunaliella* spp. responses to salt stress conditions (**Table 2**). It has been proposed that calcium-sensing receptors are important regulators of extracellular calcium content in which increases cytosolic Ca^2+^ concentration in stress conditions (Zhao et al., 2015). The co-occurrence in the meta-gene list as well as verification by machine-learning algorithms and network analysis of the calcium signal transduction gene TEF2 and lipid biosynthesis–related genes suggests that there may be potential cross-talking between Ca^2+^ signal transduction, lipid accumulation, and ROS signaling pathways in salt stress conditions. Similar cross-talking has been proposed for nitrogen starvation; so, it is feasible that similar pathways could be used for the salt stress responses also (Chen et al., 2014).

### Transport and Assimilation of Nitrogen Are Important Coordinators for Adaptation Network

Excessive cytosolic NaH_4_^+^ concentration can induce the accumulation of ROS, oxidative damages, and subsequent membrane disruption in different eukaryotic cells (Shahriari Ahmadi et al., 2013). Flexibility in NaH_4_^+^ uptake mechanisms was proposed as one of the important acclimatization approaches in salt stress conditions (Abouelsaad et al., 2016). Among the different NaH_4_^+^ transporters and assimilation-related genes that were differentially expressed in that salt stress condition (**Figure 1**), AMT1A, NIT1, NII1, NAR3, NAR4, encoding ammonium transporter, nitrate reductase, nitrite reductase, and high-affinity nitrate transporter, respectively, were selected as meta-genes (**Table 2**). Based on expression profiles, the ammonium transporter was up-regulated while the nitrate transporters and nitrate reduction genes were downregulated. A recent transcriptome from *Dunaliella viridis* shows the same expression pattern when cells are grown with NH_4_^+^ as a nitrogen source (Dums et al., 2018), which might suggests a difference in nitrogen source between the different datasets used. However, the study done with salt tolerance in tomato shows ammonium transporter up-regulation and nitrate transporter down-regulation under salt stress (Abouelsaad et al., 2016). This equally reflects that data in this study. Regulation of inorganic nitrogen metabolism genes seems to contribute to the salt stress response and possibly could be tied into crosstalk with aforementioned pathways.

## Conclusion

In conclusion, we identified a number of genes whose expression was putatively altered in the response to salt stress in two species of *Dunaliella*. The importance of identified responsive genes was validated with machine-learning algorithms, which mainly involved in ROS scavenging and signaling, chaperone-mediated autophagy, calcium signal transduction, and nitrogen metabolism. Furthermore, coordinate responses of chlorophyll a–/b–binding proteins, signaling, and ROS detoxification systems were highlighted by machine-learning and network analysis. PPI network analysis suggested the cross-talk between Ca^2+^ signal transduction, lipid accumulation, and ROS signaling pathways in salt stress conditions. Exploration of these signaling networks and additional knowledge about the identified meta-genes could provide new avenue for engineering of *Dunaliella* spp. for the production of a variety of secondary metabolites.

## Data Availability

All datasets analyzed for this study are included in the manuscript and the **supplementary files**.

## Author Contributions

Concept and design of the experiment: BP; Data analysis: BP and MF; Writing the manuscript: BP, JTD, and MAH.

## Conflict of Interest Statement

The authors declare that the research was conducted in the absence of any commercial or financial relationships that could be construed as a potential conflict of interest.
